# automRm: An R Package for Fully Automatic LC-QQQ-MS Data Preprocessing Powered by Machine Learning

**DOI:** 10.1021/acs.analchem.1c05224

**Published:** 2022-04-12

**Authors:** Daniel Eilertz, Michael Mitterer, Joerg M. Buescher

**Affiliations:** Metabolomics Core Facility, Max Planck Institute of Immunobiology and Epigenetics, Stübeweg 51, 79108 Freiburg, Germany

## Abstract

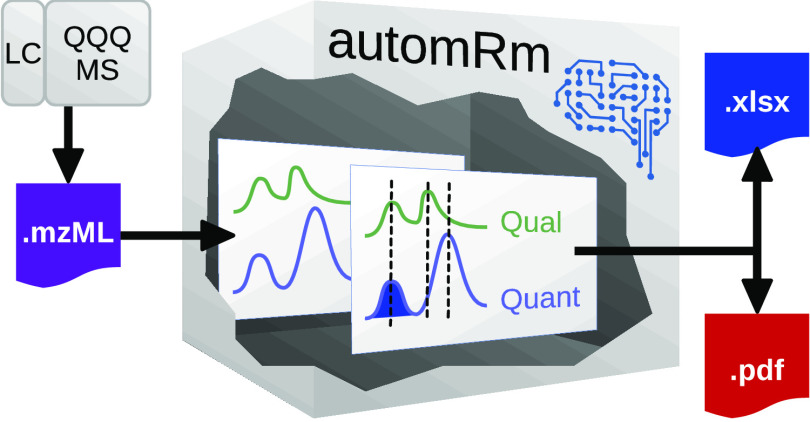

Preprocessing of liquid chromatography-mass spectrometry (LC-MS) raw data facilitates downstream statistical and biological data analyses. In the case of targeted LC-MS data, consistent recognition of chromatographic peaks is a main challenge, in particular, for low abundant signals. Fully automatic preprocessing is faster than manual peak review and does not depend on the individual operator. Here, we present the R package automRm for fully automatic preprocessing of LC-MS data recorded in MRM mode. Using machine learning (ML) for detection of chromatographic peaks and quality control of reported results enables the automatic recognition of complex patterns in raw data. In addition, this approach renders automRm generally applicable to a wide range of analytical methods including hydrophilic interaction liquid chromatography (HILIC), which is known for sample-to-sample variations in peak shape and retention time. We demonstrate the impact of the choice of training data set, of the applied ML algorithm, and of individual peak characteristics on automRm’s ability to correctly report chromatographic peaks. Next, we show that automRm can replicate results obtained by manual peak review on published data. Moreover, automRm outperforms alternative software solutions regarding the variation in peak integration among replicate measurements and the number of correctly reported peaks when applied to a HILIC-MS data set. The R package is freely available from gitlab (https://gitlab.gwdg.de/joerg.buescher/automrm).

Metabolomics aims to study the metabolism of organisms or cells by simultaneously measuring many metabolites. Metabolomics has been successfully applied in diverse fields of research such as cancer research,^1^ immunology,^2^ and environmental science.^3^ For an introduction to the topic from a user point of view, we recommend a recent review by Cholsoon and Rabinowitz.^4^

Metabolomics experiments are typically composed of four steps (Figure 1): first, metabolites are extracted from a biological specimen (tissue, cultured cells, body fluid, etc.) during sample preparation. Depending on the nature of the sample and the analytical method, additional steps might be required to further clean up the sample, concentrate the sample, or change the solvent. Second, the metabolites in the extract are quantified. Popular technologies to perform this step are gas chromatography (GC) or liquid chromatography (LC) coupled to mass spectrometry (MS) or nuclear magnetic resonance (NMR). Typically, the data is recorded in a vendor-specific file format. The third step is data preprocessing, which takes the recorded raw data as input and extracts intensity values for every feature and every metabolite. In nontargeted metabolomics, preprocessing can also include the identification of the metabolites that are represented by a feature. In the fourth step, the feature table is used to perform statistical analyses and to draw conclusions that answer the underlying experimental question.

**Figure 1:**
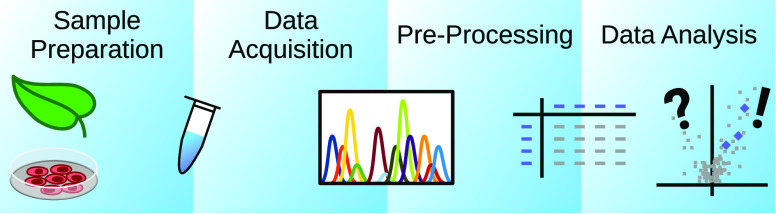
Steps of metabolomics experiments. Sample preparation includes all steps from a biological specimen to a sample that is suitable to be measured. Data acquisition uses an analytical machine to record signals representing metabolites. Preprocessing takes the acquired raw data and extracts relevant information. Data analysis links the measured information with its biological context to generate knowledge.

Metabolomics experiments can be either targeted or nontargeted. For the former, target metabolites are defined a priori and the MS machine is programmed to only acquire data for these target metabolites. Quadrupole MS is commonly used for targeted analyses because of its low cost and superior dynamic range.^5^ Typical targeted scan modes are selected ion monitoring (SIM) on single-quadrupole MS or multiple reaction monitoring (MRM) on triple quadrupole (QQQ) MS. In these modes, all other ions are physically filtered out before reaching the detector. Knowing the target metabolites allows the optimization of sample prep, chromatographic separation, and MS machine parameters for the best possible sensitivity and specificity.

Both commercial and open-source software are available for processing and analyzing metabolomics data. All vendors of MS machines also sell software for the processing and analysis of data generated on their respective machines. These software solutions vary in functionality and user-friendliness and, being closed source software, cannot be easily extended. Open-source software typically requires the conversion of the raw data from a vendor-specific format to an open format such as mzML.^6^ For most popular MS machines, this can be achieved by msconvert/ProteoWizard.^7^ Many open-source software are available for the processing of nontargeted metabolomics data or the targeted analysis of raw data acquired in full-scan mode, but only a few can handle QQQ-MS data.^8^

A major challenge in data preprocessing is that incorrect and inconsistent selection of chromatographic peaks can introduce quantitative variation on top of the well-recognized analytical and biological variation.^9^ This problem is aggravated with the increasing use of hydrophilic interaction liquid chromatography (HILIC) in metabolomics applications, which often suffers from suboptimal retention time (RT) reproducibility.^10^ Typically, some form of user intervention or user oversight is required to obtain satisfactory data processing of targeted metabolomics data.

Manual peak review greatly profits from the operator’s experience with the employed chromatographic separation and mass spectrometric detection. It encompasses several tasks for which operators typically compare multiple metabolites and samples before making a decision. For example, selecting the correct peak can be challenging when there are other, incorrect, peaks that might even have higher intensity and similar RT. Selecting the correct peak borders (start, end, baseline) can be difficult if there is a nonflat background signal. In real-world metabolomics data, this leads to many borderline decisions that greatly depend on the individual operator.

Fully automatic data preprocessing can have several advantages. It requires no expert knowledge of metabolomics technology by the operator and can thus be used by many scientists. Fully automatic processing can be performed in minutes, while manual peak review of the same data set can take hours or even days. Since computers do not make random errors, the whole process is highly reproducible. In addition, computers do not have any expectations concerning the result of an experiment and therefore do not risk introducing an expectation bias in the data. The challenge is to develop fully automatic data preprocessing that performs as well or even better than a human expert. Here, we present automRm, an R package for fully automatic preprocessing of LC-QQQ-MS data that is suitable for both HILIC and reversed-phase chromatography. Two key steps of automRm, namely, peak picking and peak reporting, rely on machine learning (ML) because it allows the recognition of complex patterns in high-dimensional input data.^11^

## Materials and Methods

We have developed and tested automRm in R version 3.6.0 on a 64-bit Linux machine. In addition, we have tested automRm in R version 4.0.2 on a 64-bit laptop running macOS 10.14.

Installation of automRm is simple from the R console using this command:

devtools::install_gitlab(’joerg.buescher/automRm@master’, host = ‘https://gitlab.gwdg.de’).

### Sample Preparation

Different concentrations of acetyl-CoA (0, 0.01, 0.1, 1 ppm) were spiked into four different matrices (Milli-Q H_2_O, commercially available fetal bovine serum (FBS), extract of HepG2 cells, extract of HEK293 cells). For Milli-Q H_2_O and FBS, 100 μL was added to 400 μL of solvent, vortexed, incubated for 5 min on wet ice, and centrifuged (3 min at 20 000*g*). Three different solvent compositions were used: 100% methanol, 100% acetonitrile, and 50:50 methanol/acetonitrile. Three different solvent compositions were used to extract cells at a concentration of 2 × 10^6^ cells/mL: 80:20 methanol/Milli-Q H_2_O, 80:20 acetonitrile/Milli-Q H_2_O, and 40:40:20 methanol/acetonitrile/Milli-Q H_2_O. Extracts were vortexed, incubated for 5 min on wet ice, and centrifuged (3 min at 20 000*g*). For HILIC chromatography, clear supernatants were transferred to PCR plates, an equal volume of ^13^C yeast extract (ISOtopic solutions) was added, and the plate was sealed with an EZpearce film and stored at −80 °C until analysis. For reversed-phase analysis, an aliquot of extract was added to an equal volume of ^13^C yeast extract, and then the mixture was dried by speedvac and resuspended in the original volume in Milli-Q H_2_O prior to transfer to PCR plates, which were sealed with an EZpearce film and stored at −80 °C.

### LC-QQQ-MS

Three different chromatographic methods were used. All targeted metabolite quantifications by LC-MS were carried out using an Agilent 1290 Infinity II UHPLC in line with an Agilent 6495 QQQ-MS operating in MRM mode (HILIC method 1) or DMRM mode (HILIC method 2 and reversed-phase method). MRM transitions were optimized separately for all compounds using pure standards or inferred from closely related compounds. Both data sets HILIC 1a and HILIC 1b were recorded using HILIC method 1 but with different sets of target compounds.

### Chromatographic Separation by HILIC Method 1 (LunaNH2)

The LunaNH2 HILIC method has been adapted from a previously published method by Bajad et al.^21^ Chromatographic separation was performed using a Phenomenex LunaNH2 column (50 × 2 mm, 3 μm particles). Buffer A was 10 mM NH_4_OH in water, and buffer B was 5 mM ammonium carbonate in 90:10 acetonitrile/water. The gradient profile was 0 min, 100% B, 1 mL/min; 0.5 min, 100% B, 1 mL/min; 4.7 min, 30% B, 0.75 mL/min; 5.1 min, 10% B, 0.75 mL/min; 7.5 min, 10% B, 0.75 mL/min, 7.8 min, 100% B, 0.75 mL/min; 8.4 min, 100% B, 1 mL/min; and stop time: 9.5 min. The injection volume was 3 μL, the column temperature was 30 °C, and the autosampler temperature was 5 °C. MS source parameters were as follows: gas temp: 200 °C, gas flow: 17 L/min, nebulizer: 60 psi, sheath gas temp: 350 °C, sheath gas flow: 11 L/min, capillary voltage: 1800 V, and nozzle voltage: 800 V. iFunnel parameters were as follows: high-pressure RF positive: 110 V, high-pressure RF negative: 90 V, low-pressure RF positive: 80 V, and low-pressure RF negative: 60 V.

### Chromatographic Separation by HILIC Method 2 (iHILIC(P))

The iHILIC(P) method has been adapted from a previously published method by Chaleckis et al.^22^ Chromatographic separation was performed using a HILICON iHILIC(P) classic column (100 × 2 mm, 5 μm particles). Buffer A was 20 mM ammonium carbonate and 5 μM medronic acid in Milli-Q H_2_O and buffer B was 90:10 acetonitrile/buffer A. The gradient profile was 0 min, 95% B, 120 μL/min; 18 min, 55% B, 120 μL/min; 19 min, 20% B, 120 μL/min; 21.5 min, 20% B, 120 μL/min; 22 min, 95% B, 120 μL/min; 23.5 min, 95% B, 120 μL/min, 25.5 min, 95% B, 300 μL/min; and stop time: 30 min. The injection volume was 2 μL, the column temperature was 40 °C, and the autosampler temperature was 5 °C. MS source parameters were as follows: gas temp: 240 °C, gas flow: 15 L/min, nebulizer: 50 psi, sheath gas temp: 400 °C, sheath gas flow: 11 L/min, capillary voltage: 2000 V, and nozzle voltage: 300 V. iFunnel parameters were as follows: high-pressure RF positive: 110 V, high-pressure RF negative: 90 V, low-pressure RF positive: 80 V, and low-pressure RF negative: 60 V.

### Chromatographic Separation by Reversed Phase

Reversed-phase LC has been widely used in metabolomics; this method has been adapted from previous methods.^23−26^ Chromatographic separation was performed using a Waters CSH C18 column (100 × 2 mm, 1.7 μm particles). Buffer A was 0.1% formic acid in water, and buffer B was 50:50 acetonitrile/methanol. The gradient profile was 0 min, 0% B; 4 min, 0% B; 19 min, 97% B; 24.5 min, 97% B; 25 min, 0% B; 27 min, 0% B; and stop time: 27 min. The flow rate was 400 μL/min, the injection volume was 3 μL, the column temperature was 30 °C, and the autosampler temperature was 5 °C. MS source parameters were as follows: gas temp: 200 °C, gas flow: 17 L/min, nebulizer: 60 psi, sheath gas temp: 350 °C, sheath gas flow: 11 L/min, capillary voltage: 1800 V, and nozzle voltage: 800 V. iFunnel parameters were as follows: high-pressure RF positive: 110 V, high-pressure RF negative: 90 V, low-pressure RF positive: 80 V, and low-pressure RF negative: 60 V.

## Results and Discussion

### Implementation of automRm in R

**Figure 2:**
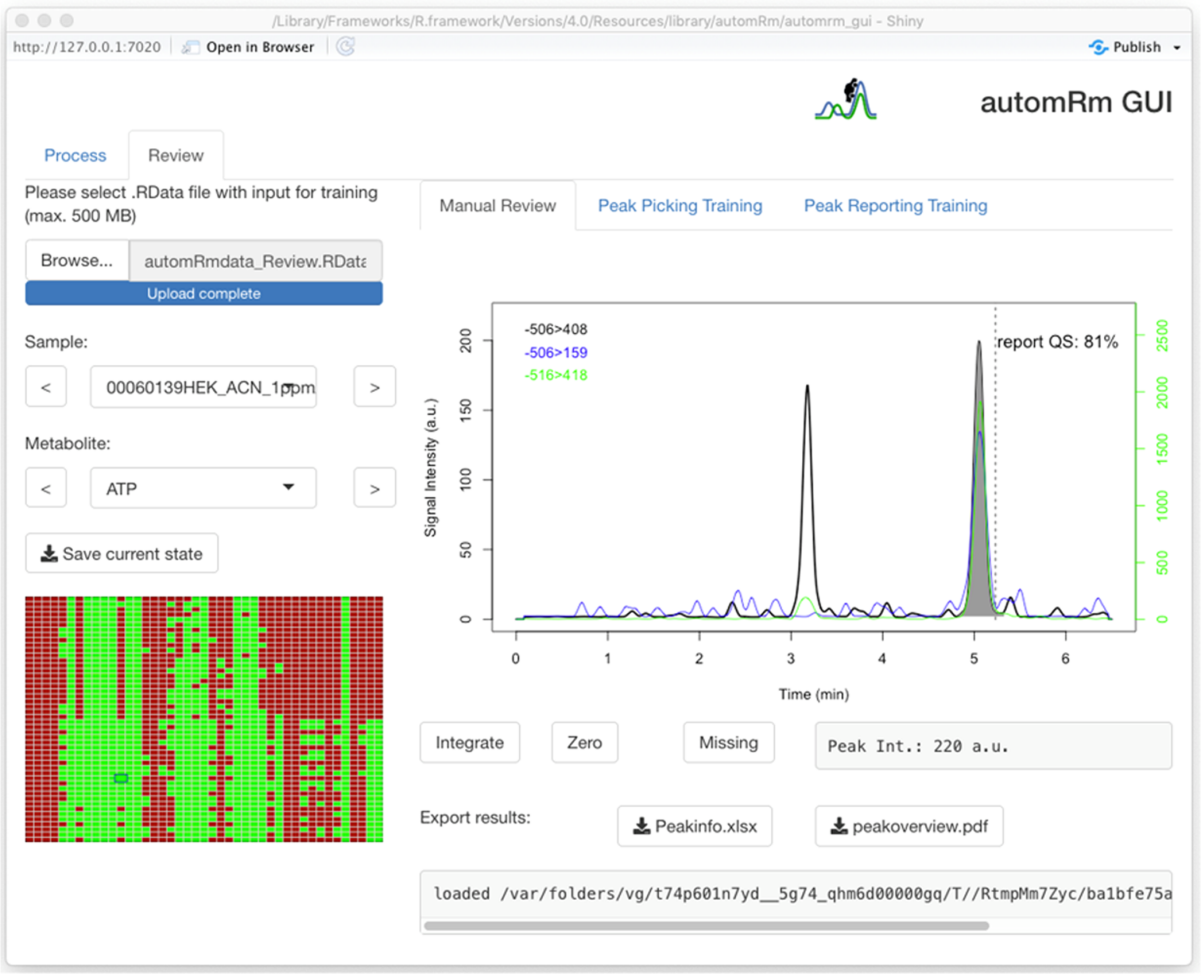
Graphical user interface (GUI) in the manual peak review mode. The left-hand panel allows loading of and navigation within data sets. The right-hand panel displays selected chromatograms and allows the modification of peak integration and export of data.

We have opted to implement automRm in the R environment^12^ because of its widespread use in the metabolomics community and the many available packages that facilitate handling of mass spectrometry data in general and metabolomics data in particular. In fact, automRm depends on several packages such as mzR^7^ for parsing of mzML files, caret^13^ for machine learning (ML), and openxlsx^14^ for reading and writing of xlsx files. The standard output of automRm is tailored for scientists who are not experts in metabolomics or R and therefore comes in xlsx and pdf formats. In addition, the results generated by automRm can easily be used within R to perform advanced statistical analyses or generate additional plots. There are two main functions in automRm: process_batch() for preprocessing of batches of raw data and train_model() for training the ML models used in process_batch(). The complete functionality of automRm including training of ML models, batchwise preprocessing of raw data, and manual peak review is also available through a graphical user interface automrm_gui() (Figure 2).

The source code of automRm including a detailed manual and description of the main functions in plain English is available online (https://gitlab.gwdg.de/joerg.buescher/automrm). Example data sets including input files for the training of ML models, pretrained ML models, and data preprocessing output files in xlsx and pdf formats are available online (https://gitlab.gwdg.de/joerg.buescher/demodata). Video tutorials for the use of automRm with command line interface and GUI are available online (https://vimeo.com/681364369 and https://vimeo.com/681366086).

### Batchwise Preprocessing

**Figure 3:**
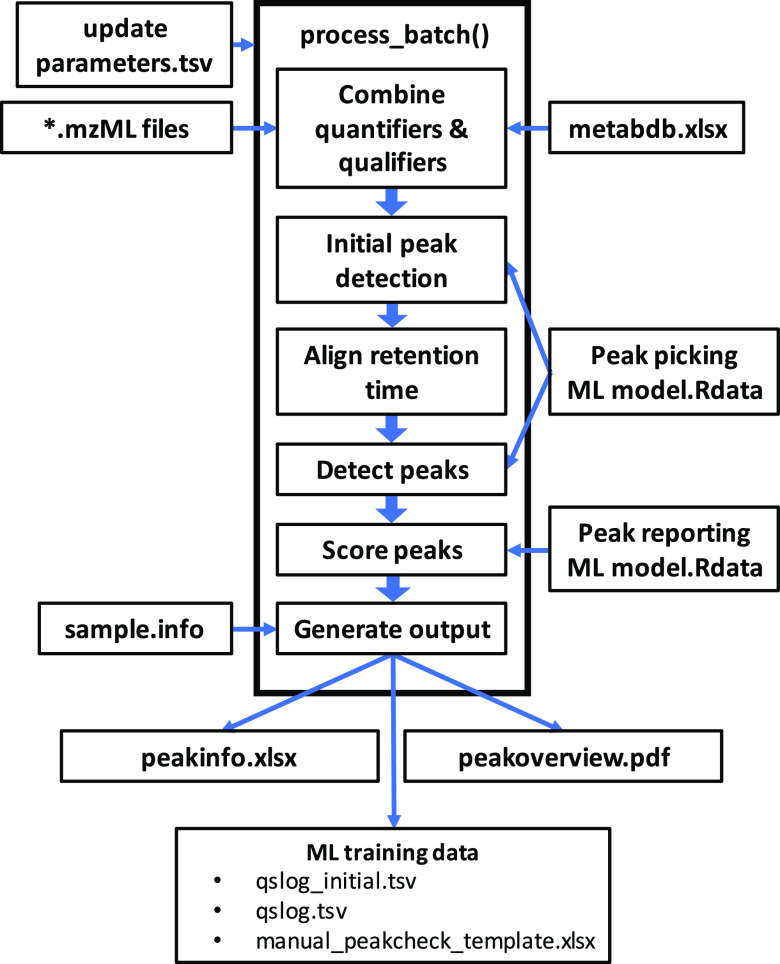
Flowchart of the process_batch() function. Thin arrows indicate the reading of input files and writing of output files. Thick arrows illustrate the chronological order of subroutines used for peak picking and peak evaluation.

Preprocessing of raw data occurs in a series of steps (Figure 3). Initially, user-defined processing parameters such as the location of raw data files, the location of the saved ML models, and the location of the xlsx file with metabolite-specific settings are parsed from a tsv file (update_prm.tsv). Next, raw data is parsed from mzML files and additional metabolite-specific values are parsed from an xlsx file (metabdb.xlsx). These additional values include the traces to use as quantifiers and qualifiers for a given metabolite, expected RT, expected ratio of signal intensity of quantifier and qualifier, and database IDs. Sample-specific metadata such as sample names and biological information is then parsed from a flat file (sample.info). Subsequently, the initial peak picking is performed independently for each metabolite in each sample. Specifically, local maxima are detected in a smoothed version of the quantifier trace and ranked by peak height. The degree of smoothing and the number of candidate peaks to be evaluated are processing parameters that can be defined in update_prm.tsv. A total of 20 quality scores (QS) that characterize chromatographic peaks by shape, agreement between quantifier and qualifiers, and deviation from expected RT are calculated for each peak candidate (Supporting Table S1). These QS serve as input for the peak picking ML model to predict the overall peak classification score. If no ML model is present, the quality scores are averaged to obtain the classification score. The sample with the highest average of peak classification scores across all metabolites is then selected as the reference sample for RT alignment. To enable subsequent training of the peak picking ML model, QS of all detected peaks can optionally be written to a tsv file (qslog_initial.tsv) at this stage.

We have previously observed that RT shifts between two HILIC runs are typically a function of the squared RT. However, RT shifts of individual metabolites can deviate from this trend by up to 30 seconds even for metabolites that elute in close proximity. Therefore, we first calculate RT shifts separately for each metabolite in each sample by the simultaneous cross-correlation of quantifier and qualifier chromatogram with the respective chromatograms in the reference sample in a time window around the expected RT. To eliminate artifacts from improbably pronounced shifts, RT shifts of all metabolites in a sample are fitted as a quadratic function of RT. This correlation is optionally visualized (shiftplots.pdf). Subsequently, the RT shift calculation is repeated with a smaller time window around the fitted value.

To enable robust and reproducible peak detection even for noisy and low abundant signals, we sum the RT-shifted chromatograms of a metabolite across all samples. Next, peak picking is performed on the summed prototype chromatogram to determine the most likely peak candidate using the same quality scores and the same peak picking ML model that were also used for initial peak picking. The start and end of the best prototype peak are then propagated back to the chromatograms of each sample to determine the peak area and peak height. To facilitate training of the peak reporting ML model, the quality scores of all peaks can be written to a tsv file (qslog.tsv) and a template for generating the matching ground can be written to an xlsx file (manual_peakcheck_template.xlsx).

To evaluate if the peak of a given metabolite in a given sample is of sufficient quality to be reported to the user, the peak reporting ML model uses the same quality scores that were previously used by the peak picking ML model. To take into account the quality of the peak of a metabolite in a sample relative to the quality of the respective peaks in all samples, six quality scores are added to the original quality scores. Specifically, these are the classification scores from the peak picking ML model and the 0th, 25th, 50th, 75th, and 100th percentiles of these classification scores of a metabolite across all samples. The intensity values of peaks that are selected by the peak reporting ML model are written to an xlsx file (peakinfo.xlsx). Additionally, the peak height and peak area, both normalized to ^13^C qualifier or raw, are written in separate sheets as alternative representations of relative amounts of metabolites. To provide users with a visual impression of their data, all chromatograms are plotted in a pdf file (peakoverview.pdf).

### Training of ML Model

**Figure 4:**
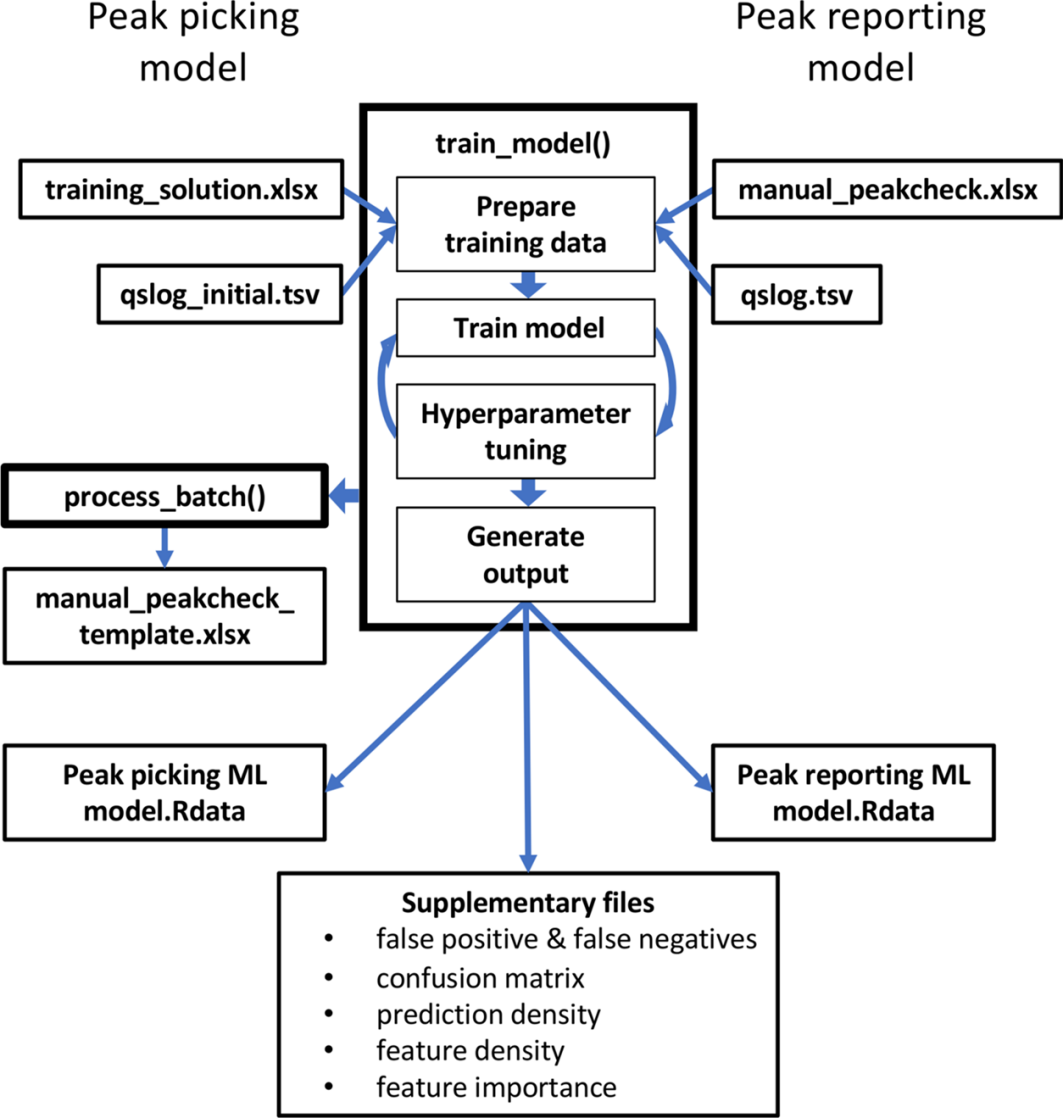
Flowchart of the train_model() function. Thin lines indicate input and output files. Thick lines illustrate the chronological order of subroutines used to train the peak picking ML model (left side) and the peak reporting ML model (right side).

The function train_model() can generate both the peak picking ML model and the peak reporting ML model from one set of raw data and two (expert-) user-generated training solutions. To generate a sufficiently large training data set for the peak picking ML model, multiple peak candidates (default = 5) are extracted for each metabolite in each sample and saved to a tsv file (qslog_initial.tsv) by process_batch() (Figure 3). Training solutions are in the form of an xlsx file (training solution.xlsx) and must provide peak area, peak start, and peak end for every metabolite in every sample. Absent peaks must be indicated by peak area = 0. All peak candidates with an apex between peak start and peak end stored in the training solution are deemed correct peaks, while all other peaks are flagged as incorrect. Training data sets are automatically split into subsets for training (80%) and validation (20%) of ML models. Subsequently, hyperparameter tuning and ML model training are performed automatically to obtain an ML model that can best discriminate between correct and incorrect peaks (Figure 4). Next, the training data is processed by process_batch() using the newly generated peak picking ML model to generate a tsv file (qslog.tsv) that contains for every metabolite in every sample the classification scores of the peak picking ML model, which are required for training of the peak reporting ML model.

Since automRm is designed to preprocess data without expert user oversight, tight quality control is required to not report erroneous or inconsistent results. The training solution must be provided in an xlsx file (manual_peakcheck.xlsx) and contain one of three possible scores for each metabolite in each sample: 0 = peak should not be reported, 1 = do not use for training, and 2 = peak should be reported. To facilitate the generation of the training solution, a template file (manual_peakcheck_template.xlsx) is generated automatically by process_batch() after the peak picking ML model has been trained. Splitting of training data for training (80%) and validation (20%), hyperparameter tuning, and ML model training are then performed automatically to obtain an ML model that can best identify the peaks that should be reported.

### Impact of ML Algorithm

**Figure 5:**
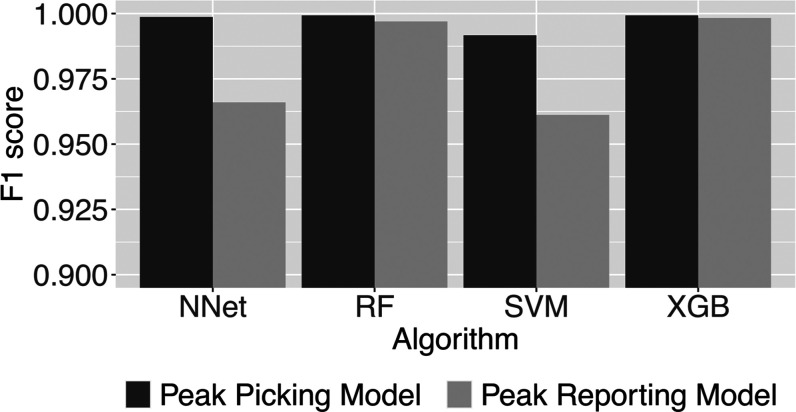
Comparison of F1 score as a measure for classification accuracy among four different ML algorithms for the peak picking task and the peak reporting task. NNet, artificial neural network; RF, random forest; SVM, support vector machine; and XGB, extreme gradient boosting.

To determine the impact of the employed ML algorithm, we have tested the speed and performance of four popular ML algorithms available in the caret package,^13^ namely, artificial neural networks (NNet), random forest (RF), support vector machine (SVM), and extreme gradient boosting (XGB) (Figure 5). As a measure of ML model accuracy, we have computed the F1 score, which combines precision and recall. For all algorithms, we have separately optimized ML model accuracy by hyperparameter tuning. For this test, we have used the combined data set of 48 samples measured with four LC-QQQ-MS methods. This data set contains 7817 positive and 32032 negative examples for peak picking and 4987 positive and 4164 negative examples for peak reporting. The ground truth was generated by manually assigning labels to all examples.

All algorithms performed very well for the peak picking task with F1 scores greater than 0.99. For the peak reporting task, random forest or extreme gradient boosting performed better than neural networks and support vector machines. The former two algorithms allowed almost perfect peak classification for the peak reporting task.

**Figure 6:**
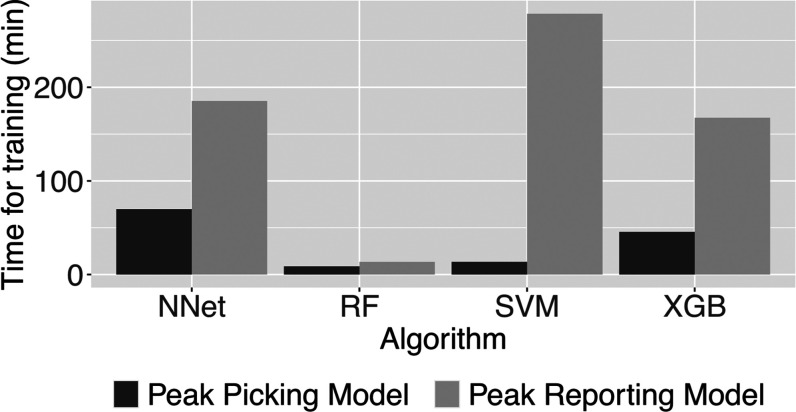
Comparison of the time required for training of ML models on a consumer-grade laptop. NNet, artificial neural network; RF, random forest; SVM, support vector machine; and XGB, extreme gradient boosting.

While testing the accuracy of the different ML algorithms, we have noted that the time required for training differed dramatically between algorithms and classification tasks (Figure 6). Training of the peak picking ML model was faster than that of the peak reporting ML model despite the larger training data set of the former. Of note, random forest (RF) was the fastest ML model to train for either task. Of note, there was hardly any difference in the time required for the application of the ML models in process_batch() (data not shown).

### Impact of Training Data Set

**Figure 7:**
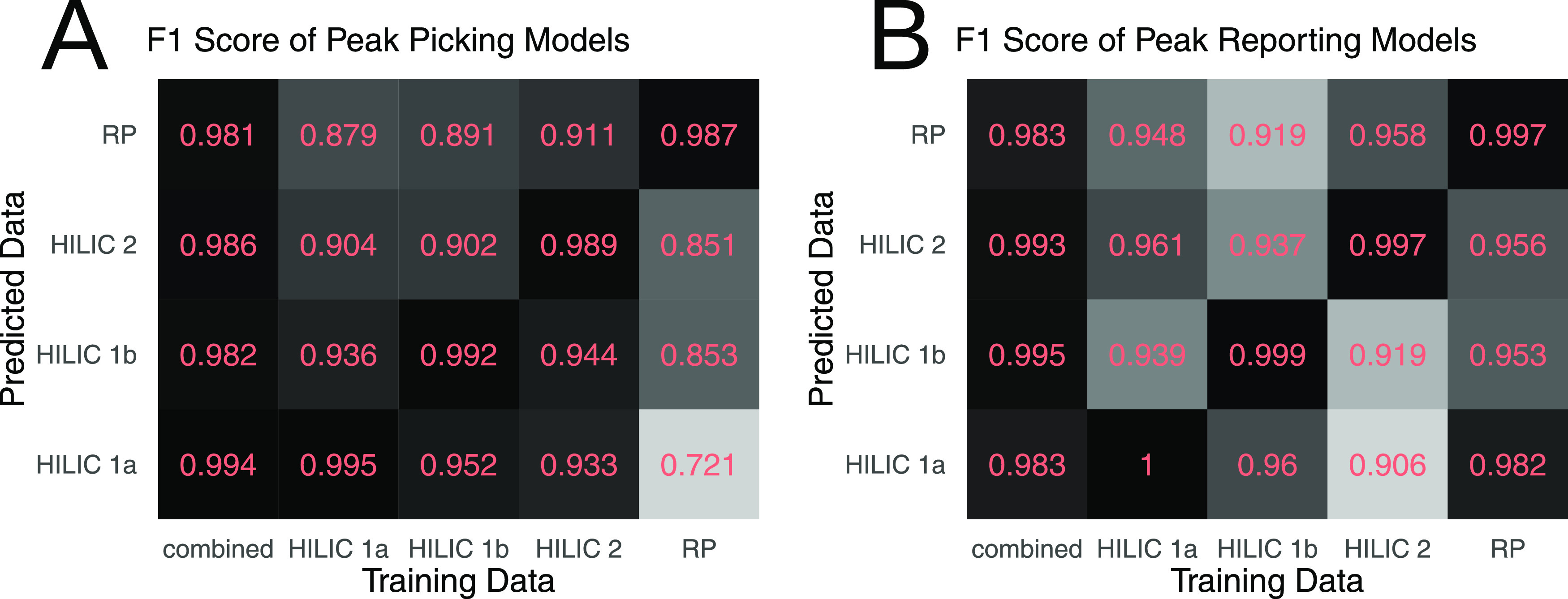
F1 score as a measure of classification accuracy of (A) peak picking ML models and (B) peak reporting ML models trained on five different data sets and applied to four different data sets.

To test the impact of the training data set on the classification quality, we have trained separate peak picking ML models and peak reporting ML models for each of the training data sets and on the combined data set that includes all four training data sets (Figure 7). We have opted to use the random forest algorithm for this test because of its superior combination of classification fidelity and speed. As expected, specialized classification ML models that were trained on only one data set perform best for the data set that they were trained on. Peak picking ML models trained on one HILIC data set performed better on other HILIC data sets than the peak picking ML model that was trained on the reversed-phase data set (Figure 7A). Interestingly, such a similarity among ML models trained on HILIC data sets was absent among peak reporting ML models. For example, the peak reporting ML model trained on the reversed-phase data set performed almost as well as the peak reporting ML model trained on the combined data set when used on the HILIC 1a data (Figure 7B). The more general classification ML model that was trained on the combined data set delivered the second-best performance for every single data set with F1 scores larger than 0.98 in every case. This rendered the combined model most useful for general use with any (future) data set.

**Figure 8:**
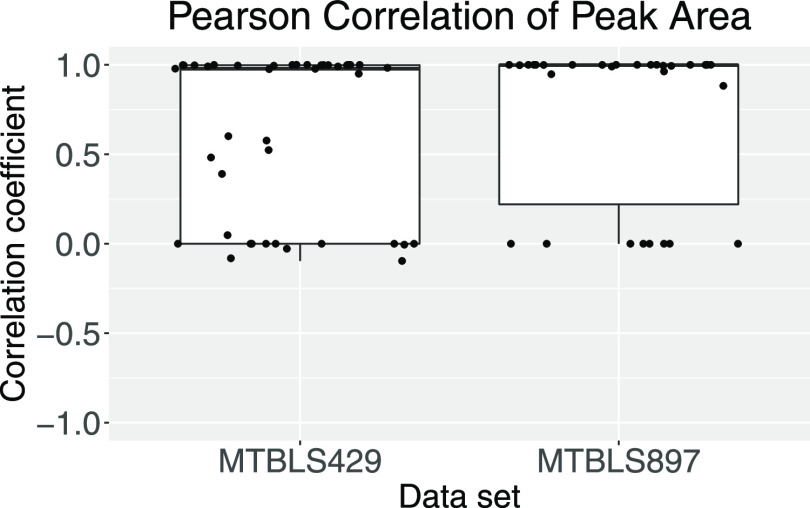
Distribution of Pearson correlation coefficients between peak areas determined by automRm and published peak areas.

As the ultimate test of data preprocessing performance on independent data sets, we have used automRm with the “combined” peak picking and peak reporting ML models on public data sets from Metabolights.^15^ Data set MTBLS429 contains measurements of pyrrolizidine alkaloids from leaf surfaces and leaf tissues using reversed-phase chromatography on a C18 column coupled to QQQ-MS detection.^16^ Data set MTBLS897 contains measurements of secondary metabolites from grapes using reversed-phase chromatography on a C30 column coupled to QQQ-MS.^17^ To evaluate the overall performance, we correlated the signal intensity determined by automRm to that reported by the authors of the two data sets (Figure 8). In addition, we have compiled some illustrative examples from publicly available data sets that demonstrate the performance and limitations of fully automatic preprocessing by automRm (Supporting Table S2).

The signal intensity as measured in the peak area that had been determined by automRm was in very good agreement (Pearson correlation >0.95) with the signal intensity values published by the authors of the data sets for 22 of 34 and 20 of 30 metabolites in data sets MTBLS429 and MTBLS897, respectively. The remaining metabolites fell into one of two categories: (1) Measurements that had been filtered out by automRm because of insufficient signal quality (indicated by correlation coefficient = 0 in Figure 8). Concretely, we have trained the peak reporting ML model to remove very low abundant measurements and metabolites with only a quantifier trace but no qualifier trace. (2) Metabolite peaks that were in close proximity to a much larger peak in both quantifier and qualifier traces. Of note, these results were achieved with ML models that had been trained mostly on HILIC data in which chromatographic peaks frequently shift between samples and therefore deviations from the expected RT commonly occur. With appropriate training of the ML models on data that is more similar to these public data sets, even better agreement between the automRm output and the originally reported signal intensities can be expected.

### Impact of ML Quality Scores

**Figure 9:**
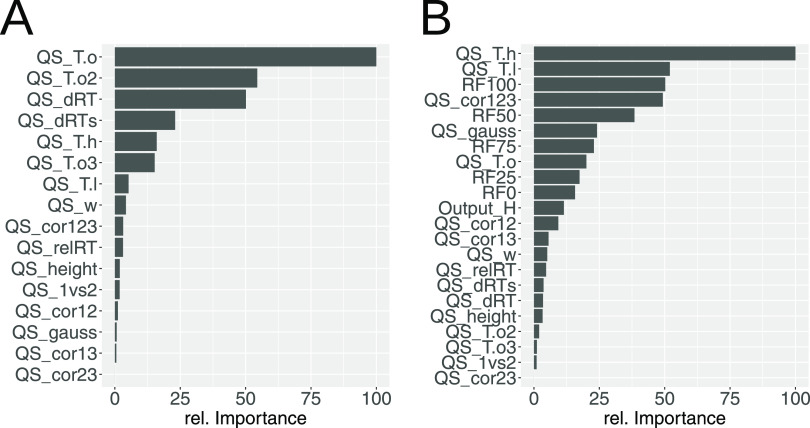
Relative feature importance in random forest ML models for peak picking and peak reporting. Interestingly, the same quality score can have drastically different importance in the two models. For a detailed description of quality scores, see Supporting Table S1. (A) Peak picking ML model and (B) peak reporting ML model.

We have compared the relative importance of features for the peak picking ML model and the peak reporting ML model using our combined data set and random forest ML models (Figure 9). QS representing the height of a peak relative to its surrounding had great importance for both ML models (for example, QS_T.o: ratio of the quantifier peak height to the highest point outside the peak, QS_To2: like QS_T.o but for the qualifier trace, QS_T.h: ratio of the qualifier peak height to the height of the higher peak border, QS_T.l: ratio of the qualifier peak height to the lower peak border). For the peak picking model, QS representing the deviation from the expected RT (QS_dRT: deviation from the expected RT, QS_dRTs: deviation from the expected RT taking into account the predicted peak shift relative to the reference sample) were also important (3rd and 4th ranks). Interestingly, they only had little influence in the peak reporting ML model. QS that exhibited great importance in the peak reporting ML model, but not in the peak picking ML model, describe the shape of a peak (for example, QS_cor123: correlation among quantifier and qualifier traces, QS_gauss: correlation of the quantifier peak with Gauss peak). QS representing the output of the peak picking model (output_H: score calculated by the peak reporting ML model for a metabolite in a sample, RF0 to RF100: quantiles of output_H values of a metabolite across all samples) are only used in the peak reporting ML model and were found to be very important for this model.

### Comparison to Other Data Preprocessing Solutions

**Figure 10:**
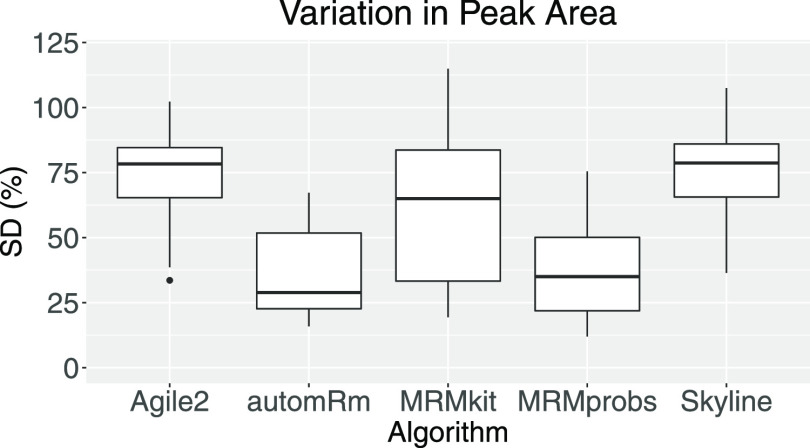
Comparison of the standard deviation of groups of replicates in the HILIC 1a data set processed with different algorithms.

To compare the performance of automRm to alternative solutions for LC-QQQ-MS data preprocessing, we have processed our HILIC 1a data set using automRm with the combined random forest ML models for peak picking and peak reporting, MassHunter 8 with Agile2, MRMkit,^18^ MRMprobs,^19^ and Skyline^20^ (Figure 10). MassHunter and Skyline were designed to facilitate manual peak review; however, we have opted to only compare the automatically generated results retaining all reported peaks irrespective of flags. As a measure of how reproducibly peaks were detected, we compared the standard deviation (SD) of peak area among replicates (12 groups of quadruplicates) for the 31 metabolites that could be processed in all five software solutions (Figure 10).

MassHunter/Agile2 and Skyline reported a peak for almost every metabolite in every sample and left it to the user to remove missing signals and correct erroneous peak detections. Consequently, the relative SD among replicates in the automatically generated output was high. MRMkit also reported peaks for every metabolite in every sample but achieved more consistent peak integrations across samples indicated by a lower average SD among replicates. MRMprobs and automRm obtained similar SD among replicates with a slight advantage for automRm.

Next, we assessed the ability of the different software solutions to discriminate between peaks that correspond to the target metabolite and should thus be reported (correct peaks) and all other signals. A given chromatogram can contain zero or one correct peak, and, due to the limited specificity of MS detection, it might contain additional (incorrect) peaks. Ideally, data preprocessing reports the signal intensity of all correct peaks and reports missing or zero if there is no correct peak. We manually reviewed our HILIC 1a data set to define all correct peaks and then classified the output of the different software for every metabolite in every sample as one of the following: (1) true positives (TPs): the correct peak was reported, (2) true negatives (TNs): there is no correct peak and none was reported, (3) false positives (FPs): an incorrect peak was reported in the presence or absence of a correct peak, (4) false negatives (FNs): a correct peak is present, but no peak was reported (Table 1). From these numbers, we then calculated the accuracy and specificity of the classification.

**Table 1:** Comparison of Number of Peaks That Were Correctly or Incorrectly Reported or Not Reported for Data Set HILIC 1a in Comparison to the Expert Review of the Data Set

	Agile2	automRm	MRMkit	MRMprobs	Skyline
true positive	875	887	842	820	870
true negative	1	530	0	366	0
false positive	612	1	646	201	618
false negative	0	70	0	101	0
accuracy	0.589	0.952	0.566	0.797	0.585
specificity	0.002	0.998	0.000	0.646	0.000

Both MRMprobs and automRm used multiple peak characteristics to determine if a peak is of sufficient quality to be reported and thus achieve higher accuracy and much higher specificity than the alternative software solutions. In our comparison, automRm outperformed MRMprobs in the number of correctly reported peaks as well as in the accuracy and the specificity of the classification.

## Conclusions

Fully automatic preprocessing of LC-QQQ-MS data in automRm compares favorably to alternative automatic software solutions and is faster and more convenient than manual peak review (the current standard in the field). The use of machine learning enables complex automatic decisions that surpass classical rule-based approaches. This provides the possibility to tune the quality of the reported signals to the required level of data reliability. With sufficient training on relevant raw data, results from automRm can reach a similar level of quality as manual peak review.

For optimal performance, automRm requires the use of at least one qualifier in addition to the quantifier. We use our LC-QQQ-MS platform for high-confidence measurements and therefore use two qualifiers (one unlabeled and one ^13^C labeled) wherever possible. However, the addition of qualifiers to the acquisition method reduces the number of analytes that can be detected simultaneously in the MRM mode.

In our hands, preprocessing of raw data is no longer a bottleneck in our LC-QQQ-MS pipeline since we have started to use automRm routinely. We only retreat to manual peak review for selected metabolites in special cases such as excessively dirty samples, very-low-input samples, or new LC methods.

## Supporting Information Available

Quality scores in automRm (Table S1) and example chromatograms from publicly available data sets that demonstrate the performance and limitations of fully automatic data preprocessing by automRm (Table S2) (PDF)
