# Supplementary material for: automRm: An R Package for Fully Automatic LC-QQQ-MS Data Preprocessing Powered by Machine Learning
Source: Anal Chem. 2022 Apr 12;94(16):6163–71. doi: 10.1021/acs.analchem.1c05224 (PMC9047440; doi:10.1021/acs.analchem.1c05224)
Supplement: Supplementary file 1 — ac1c05224_si_001.pdf [file ac1c05224_si_001.pdf]

Supplementary Material to

automRm: An R package for fully automatic LC-QQQ-MS data pre-processing powered by machine learning

Daniel Eilertz, Michael Mitterer, and Joerg M. Buescher*

Metabolomics Core Facility
Max Planck Institute of Immunobiology and Epigenetics
Stübeweg 51
79108 Freiburg
Germany
buescher@ie-freiburg.mpg.de

Table of Contents

Supplementary Table S 1: Quality Scores in automRm...page S-2

Supplementary Table S 2: Example chromatograms from publicly available data sets ....page S-6

Supplementary Table S 1: Quality Scores in automRm

QS_cor123	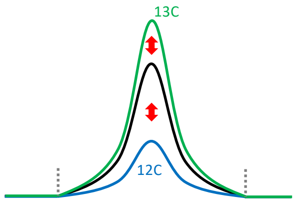	Averaged correlation of quantifier and up to 2 qualifiers within peak borders.
QS_T.o	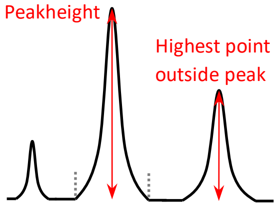	Ratio of peak top to highest point outside the peak in quantifier chromatogram.
QS_T.h	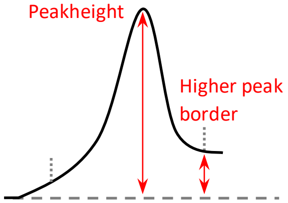	Ratio of peak top to the higher peak border.
QS_T.l	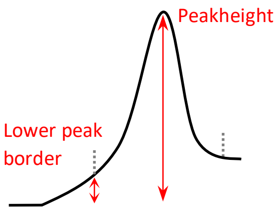	Ratio of peak top to the lower peak border.

QS_dRT	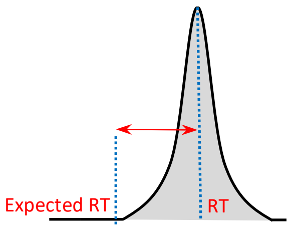	Deviation between measured and expected retention time.
QS_w	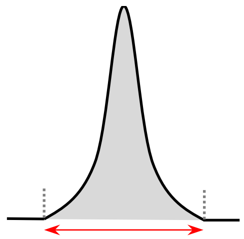	Peak width.
QS_cor12	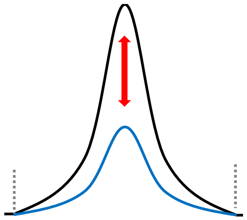	Correlation of quantifier to unlabeled qualifier.
QS_cor13	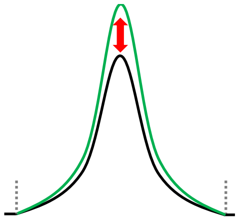	Correlation of quantifier to 13C labeled qualifier.
QS_cor23	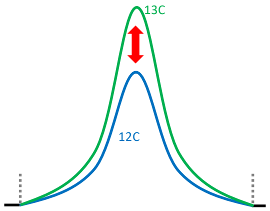	Correlation of unlabeled qualifier to 13C labeled qualifier.

QS_height	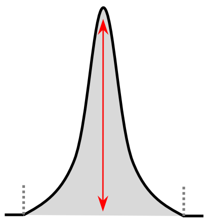	Log10 of peak height.
QS_relRT	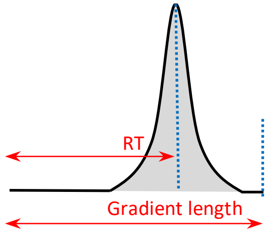	Ratio of retention time to total length of chromatographic gradient.
QS_T.o2	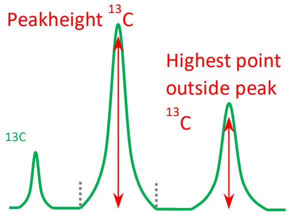	Ratio of peak top to highest point outside the peak in unlabeled qualifier chromatogram.
QS_T.o3	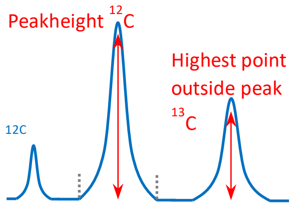	Ratio of peak top to highest point outside the peak in 13C labeled qualifier chromatogram.

QS_gauss	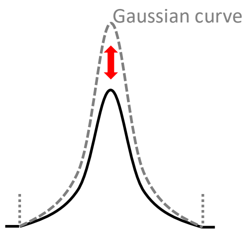	Correlation of peak to gaussian curve.
QS_1vs2	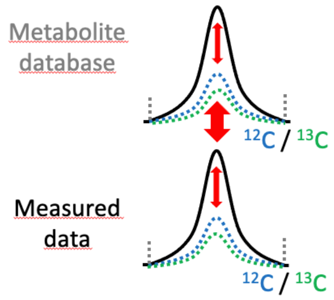	Ratio of measured and expected ratios of signal intensity of quantifier and unlabeled qualifier.
QS_dRTs	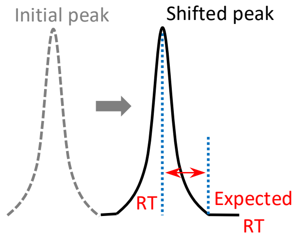	Deviation between measured and expected retention time after peak alignment.

Supplementary Table S 2: Example chromatograms from publicly available data sets that demonstrate the performance and limitations of fully automatic data pre-processing by automRm. Please note that the performance of automRm is highly dependent on the data that has been used for training of the machine learning (ML) models and the user input during training. For the generation of these examples, automRm had been trained mostly on HILIC data.

Metabolite	Sample and Dataset	Chromatogram	Comments
Acetyl-CoA	00047975TP36_3Pccm in MTBLS2145 [1]	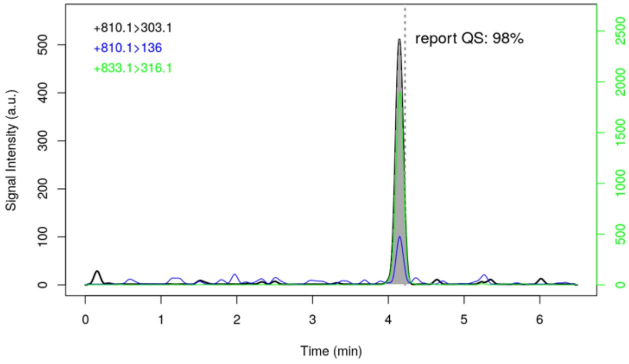	Only a single clear peak with little background and good correlation among quantifier and qualifiers result in very high quality score.
Acetyl-CoA	00047976TP48_1Pccm in MTBLS2145 [1]	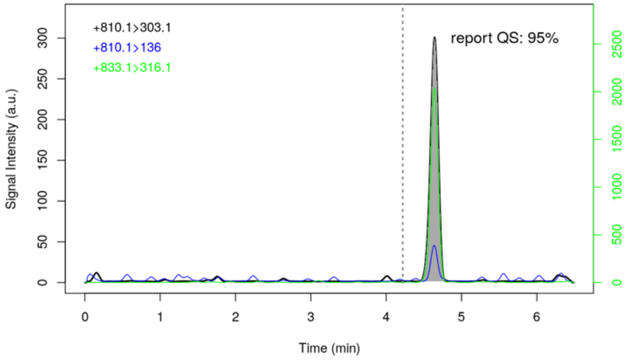	Despite a shift in retention time of about half a minute the peak is still recognized with high confidence.

Acetyl-CoA	00047966TP00_3Pccm in MTBLS2145 [1]	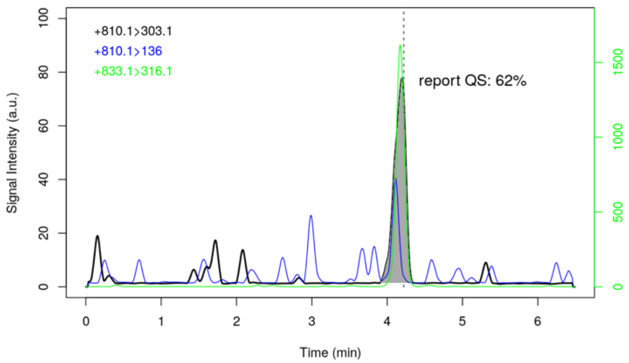	Even a very small peak is still recognized and of sufficient quality to be reported.
Acetyl-erucifoline N-oxide	P1_100730_PAs_009 in MTBLS429 [1]	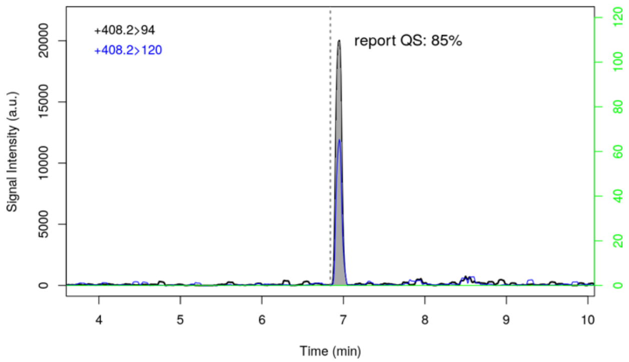	Only a single clear peak with little background and good correlation among quantifier and qualifiers but without a second qualifier results in a moderately high quality score. Of note, most metabolites in the training data set had two qualifiers.
Acetyl-erucifoline N-oxide	P1_100730_PAs_015 in MTBLS429 [1]	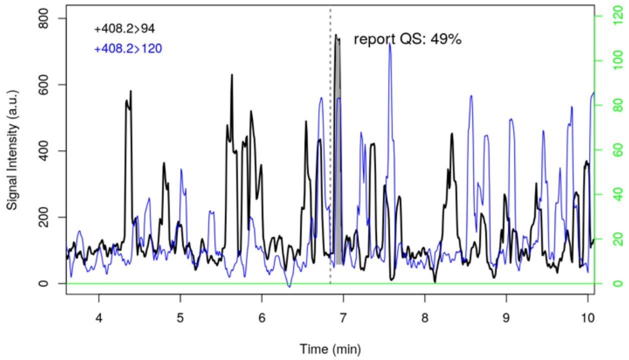	A peak that is close to background is not reported by automRm because of insufficient quality. However, the authors of the original study did report an intensity value.

Dehydro-jacoline	P1_100730_PAs_041 in MTBLS429 [1]	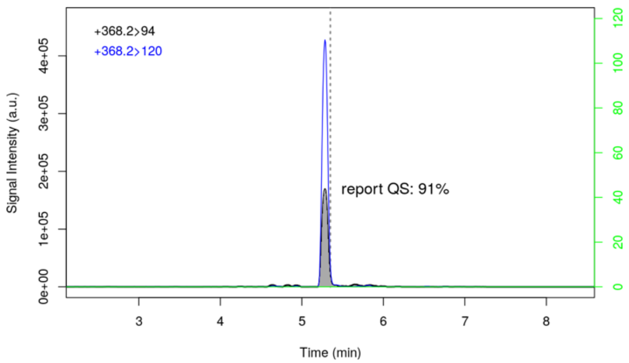	A peak is reported with high quality score by automRm, however the authors of the original study reported zero.
Dehydro-jacoline	P1_110513_PAs_006 in MTBLS429 [1]	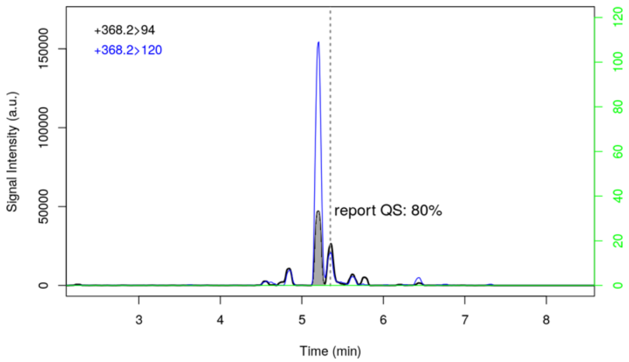	A peak is reported with moderately high quality score by automRm, however the authors of the original study reported the smaller peak that better matches the expected retention time (dotted vertical line).
Epigallo-catechin	T032_TO130_MRM in MTBLS897 [1]	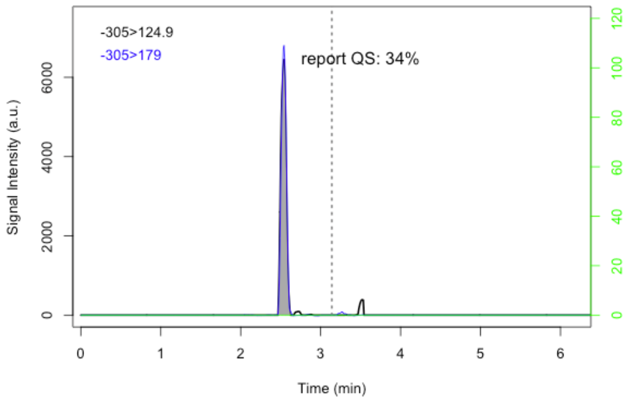	This peak was not reported by automRm because of insufficient quality. It is likely, that the deviation from the expected retention time by almost 1 min severely reduced the quality score. However, the authors of the original study did report this peak.

Fumaric acid	00047980TP60_2Pccm in MTBLS2145 [1]	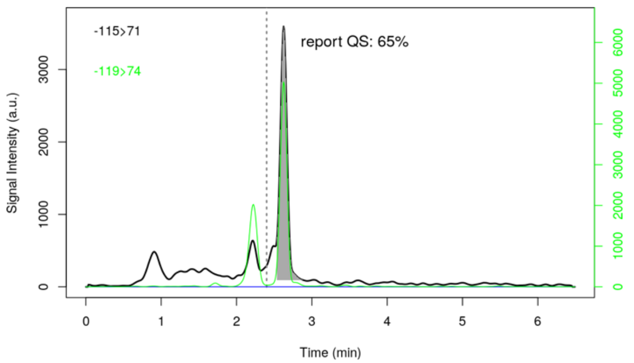	The peak is integrated without the shoulder on the left-hand side in agreement with the peak shape of the qualifier.
Glycerol phosphate	00047968TP12_2Pccm in MTBLS2145 [1]	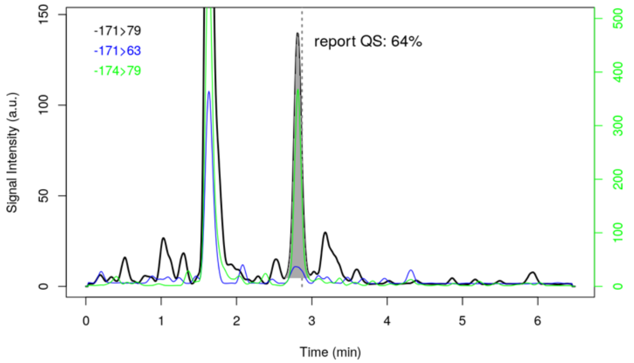	This rather small peak is correctly reported despite the presence of a much larger peak at 1.7 min that contains both qualifiers.
Glutathione reduced (GSH)	00047977TP48_2Pccm in MTBLS2145 [1]	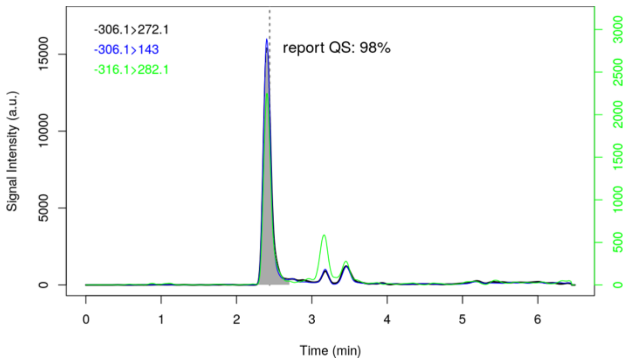	Both glutathione peaks are integrated with very similar width despite different alternative peaks in the vicinity.

Glutathione reduced (GSH)	00047979TP60_1Pccm in MTBLS2145 [1]	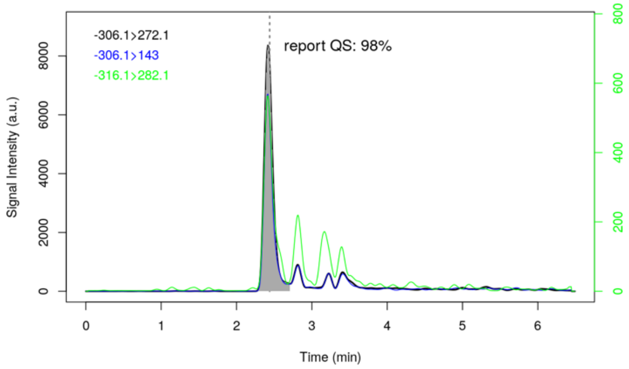	
Leucine	00060177HEK_MeOH_ACN in HILIC1b [2]	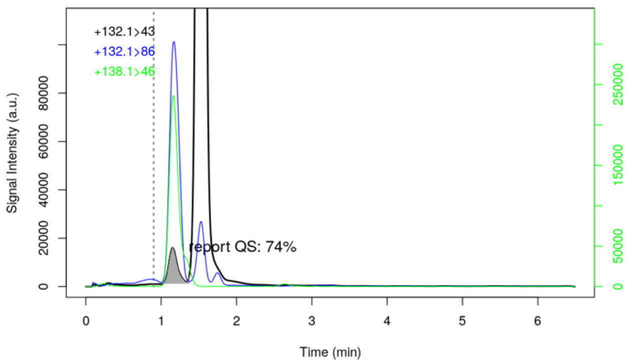	This peak is reported by automRm despite a much larger peak in the quantifier trace in its immediate vicinity. Note that the peak at 1.7 min lacks the second qualifier.
Trans epsilon viniferin	T034_TO117_MRM in MTBLS897 [1]	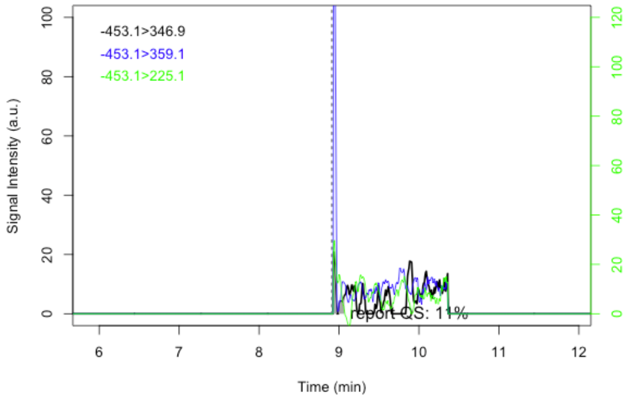	This peak is not reported by automRm because of insufficient quality. However, the authors of the original study did report an intensity value.

1 available online at https://www.ebi.ac.uk/metabolights/

2 available online at https://gitlab.gwdg.de/joerg.buescher/demodata
